# Investigating influence factors of traffic violations at signalized intersections using data gathered from traffic enforcement camera

**DOI:** 10.1371/journal.pone.0229653

**Published:** 2020-03-04

**Authors:** Chuanyun Fu, Hua Liu

**Affiliations:** 1 School of Transportation and Logistics, Southwest Jiaotong University, Chengdu, China; 2 National United Engineering Laboratory of Integrated and Intelligent Transportation, Southwest Jiaotong University, Chengdu, China; 3 National Engineering Laboratory of Integrated Transportation Big Data Application Technology, Southwest Jiaotong University, Chengdu, China; Tongii University, CHINA

## Abstract

To effectively reduce traffic violations that often cause severe crashes at signalized intersections, exploring their contributing factors seems hugely urgent and essential. This study attempted to investigate the influence factors of wrong-way driving (WWD), red-light-running (RLR), violating traffic markings (VTM), and driving in the inaccurate oriented lane (DIOL) at signalized intersections by using data collected from traffic enforcement camera in Hohhot, China. To this end, an ordinary multinomial logit model was developed. By considering the unobserved heterogeneity between observations, a random effects multinomial logit model was proposed as well. After that, the marginal effects of explanatory variables were computed. The outcomes showed that non-local vehicles were more likely to commit WWD and VTM than local vehicles. WWD and RLR frequently occurred in the daytime and evening (6:00–23:59), and on most days within a week. RLR and DIOL mainly happened in June and July. The left-turn lane ratio significantly increased RLR and DIOL. The cloudy, partly cloudy, and rainy days obviously increased WWD and VTM. The temperature from 21 to 30 degrees centigrade was apparently associated with the higher likelihoods of RLR and DIOL. According to the findings of this study, some intervention measures, targeting different vehicle types and considering temporal factors, road, and weather conditions, were recommended to reduce WWD, RLR, VTM, and DIOL at signalized intersections.

## Introduction

Intersection safety has been becoming an international concern. In Australia, almost 33% of major casualties are caused by intersection crashes [1]. In China, approximately 30% of total road fatalities happen at intersections [2]. Such serious intersection-related crashes are a result of complex interactions between road user behaviors, vehicle factors, geometric road characteristics, and environmental factors [3, 4], and primarily attributed to traffic violations, especially at signalized intersections. For example, red-light-running (RLR) is the primary cause of accidents at signalized intersections. Specifically, RLR had resulted in 4,227 severe injury crashes and 789 fatalities, according to the data gathered from January 2012 to October 2012 in China [5].

Hence, without a comprehensive analysis of causal factors, any effort to implement countermeasures to prevent or mitigate the frequency of traffic violations may be misguided. If the traffic violations at signalized intersections could be decreased or controlled efficiently, then the corresponding severe injuries and fatalities would be reduced accordingly. To effectively mitigate signalized intersection traffic violations, the first critical step is to identify their significant influence factors.

RLR is one of the signalized intersection traffic violations, which has aroused extensive attention of scholars in the field of traffic safety. At present, a large number of studies have investigated the influence factors of RLR from four aspects: human, vehicle, road, and traffic environment. With respect to human factors, age [6], gender [7], occupancy [5, 8], driving record [7] are significantly related to RLR. With regard to vehicle factors, vehicle size [6, 9], speed [10], vehicle license ownership [5], whether the preceding vehicle or the vehicle on the adjacent lane passing through the intersection during yellow [11], vehicle load [12], stopping distance [13], and approach speed [13] are obviously associated with RLR. As to road factors, intersection design [5, 14], intersection width [13], number of approaches [15], road width [15], speed on crossroad [15], width of crossroad [15], road type [12], and intersection type [12] evidently affect RLR. As for traffic environment factors, approach volume [5, 16], signal timing [11], signal mounting configurations [17], red light camera [18, 19], signal countdown timer [20], light condition [12], time of day [12, 21], day of week [21], and weather condition [12, 22, 23] distinctly impact RLR.

In addition to RLR, there are some other traffic violations usually taking place at signalized intersections, such as wrong-way driving (WWD), violating traffic markings (VTM), and driving in the inaccurate oriented lane (DIOL). The WWD is usually defined as the phenomenon that a vehicle intentionally or unintentionally travels in the opposite direction of traffic flow along with the physically divided facilities such as freeways, expressways, and the corresponding ramps [24]. The VTM includes illegally changing lane, driving over the lane marking, making U-turning at the prohibited place, and so on. The DIOL refers to the situation that a vehicle travels in an inappropriate oriented lane to pass through the intersection [25]. For instance, one vehicle in the left-turn protected lane travels straightly pass through the intersection.

As to WWD, the existing literature focus on its related crashes, injuries, and interventions on freeways and their access ramps [24, 26]. In practice, the WWD also often occurs on urban roads and at the intersections, particularly at signalized intersections with the channelized approaches. Nevertheless, to the authors’ knowledge, no studies have explored the contributing factors of WWD at signalized intersections. As for VTM and DIOL, only very few studies have examined their influence factors. Based on the self-reported questionnaires, Wang et al [27] suggested that attitude, subjective norms, and perceived behavioral control obviously affected the lane change violation at urban intersections. According to the video data collected from traffic enforcement cameras at signalized intersections, Fu et al [25] classified the behaviors of DIOL and explored their influence factors. They stated that the number of vehicles in the queue, percentage of large size vehicles, time period, traffic volume, and lighting conditions evidently influenced this violation.

It is worth noting that more possible influence factors of RLR, particularly road and environment factors, require to be further explored. Furthermore, it is still unclear as to the effects of driver demography, vehicle characteristics, road, environment, and weather conditions on WWD, VTM, and DIOL at signalized intersections. In addition, some previous studies are conducted according to the self-reported data [28], observational data [21] and questionnaires [29]. These data are somewhat subjective, which may lead to some unobserved bias in the analysis results. Besides, several studies explore the influence factors of traffic violations based on the crash data [30–32]. The problem is that, as known to all, some traffic violations cause crashes, whereas some do not. Hence, the outcomes from crash data-based studies may magnify or reduce the effects of various factors on traffic violations. To address the aforementioned issues, carrying out studies on contributing factors of specific traffic violations is vital, according to the objective and comprehensive data collected from traffic control and management tools, such as traffic enforcement cameras, since the technique of video analysis [33, 34] is economical and practicable.

Accordingly, this study, which is an extension of the authors’ previous study [25], attempted to employ the data obtained from traffic enforcement camera to examine the influences of road geometric characteristics, traffic enforcement and management measures, vehicle and temporal attributes, and weather conditions on traffic violations at signalized intersections. In our dataset, there were four traffic violations, namely RLR, WWD, VTM, and DIOL. Therefore, the ordinary multinomial logit model (OMLM) was developed to uncover the effects of contributing factors to traffic violations. By considering the unobserved heterogeneity between observations, a random effects multinomial logit model was proposed as well. Simultaneously, the marginal effects of independent variables were applied to figure out the extent to which a particular factor affected traffic violations.

## Data collection

### Intersection selection

In order to adequately reveal the effects of influence factors on traffic violations at signalized intersections, the following criteria were adopted:

- Selected intersections should be located in different areas of the city[5].
- The layout of each signalized intersection should be different.
- The traffic management and enforcement facilities should be set.
- There should be channelization at each intersection approach.

**Fig 1:**
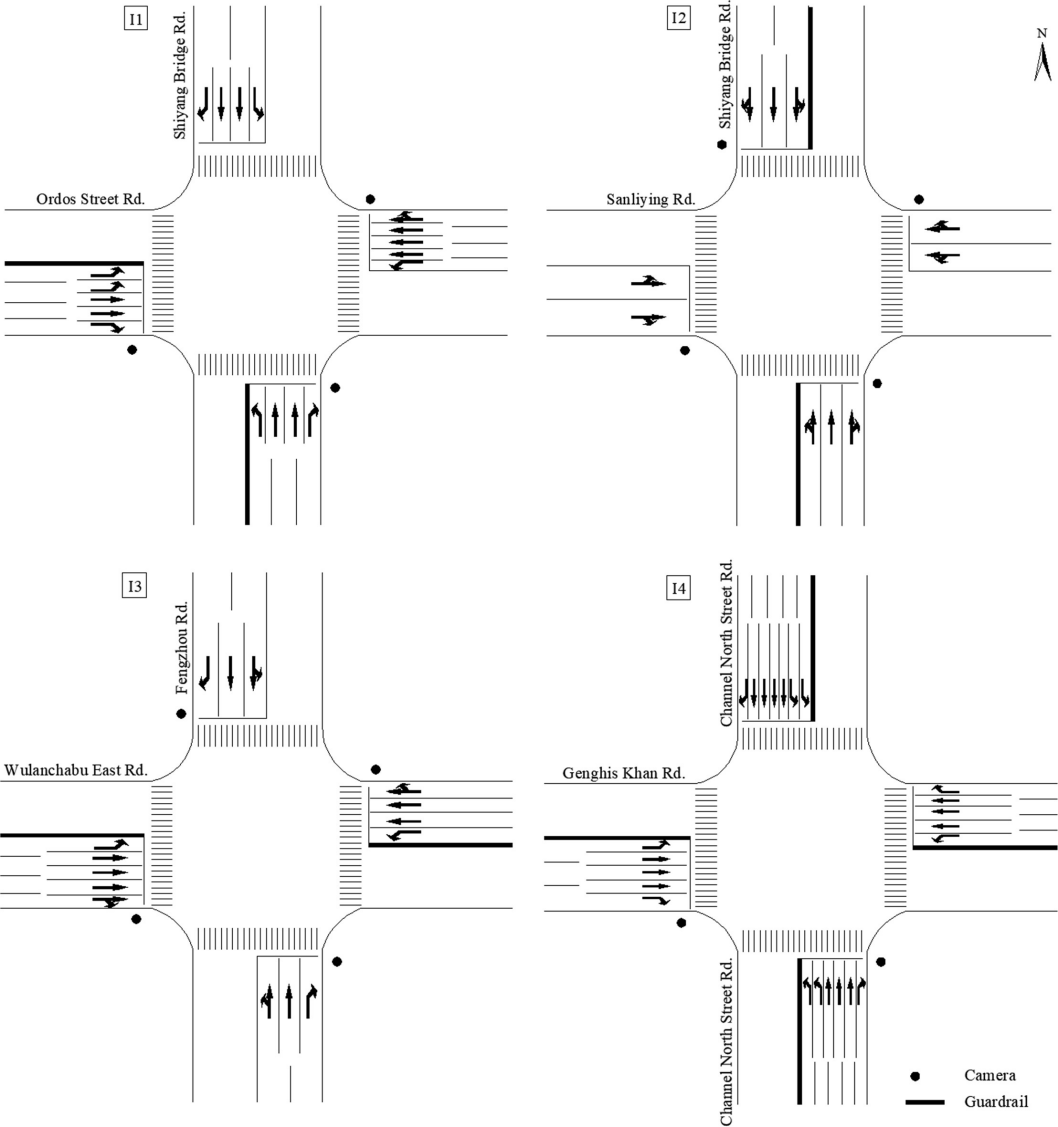
Layouts of the four selected signalized intersections.

**Fig 2:**
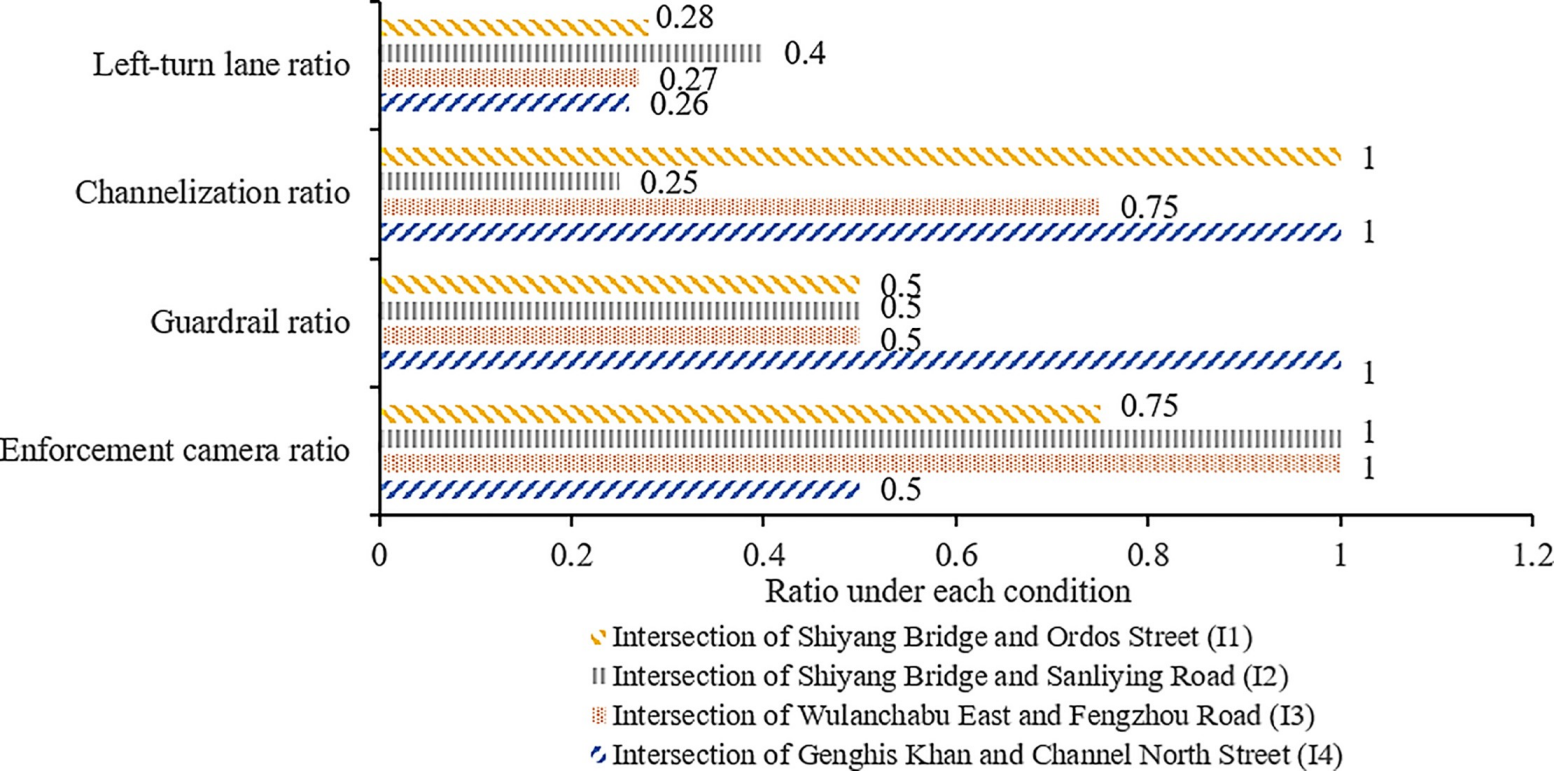
Ratios of road and traffic management conditions at each signalized intersection.

Accordingly, four signalized intersections in Hohhot, China, were selected. Their layouts are illustrated in Fig 1. The specific characteristics of these intersections are presented in Table 1 and Fig 2. It is observed that I2 and I4 have the smallest and highest number of lanes at each approach, respectively, while the ratios of left-turn lanes and enforcement cameras at I2 are higher than that at I4. However, the ratios of channelization and guardrail at I4 are higher than those at I2.

**Table 1:** Characteristics of four selected signalized intersections.

Characteristics	Four Selected Signalized Intersections			
	I1	I2	I3	I4
**Number of Lanes**				
north approach	LTL: 1	SLL: 1	SLL: 1	LTL: 2
	TL: 2	TL: 1	TL: 1	TL: 4
	RTL: 1	SRL: 1	RTL: 1	RTL: 1
south approach	LTL: 1	SLL: 1	SLL: 1	LTL: 2
	TL: 2	TL: 1	TL: 1	TL: 3
	RTL: 1	SRL: 1	RTL: 1	RTL: 1
east approach	LTL: 1	SLL: 1	LTL: 1	LTL: 1
	TL: 3		TL: 2	TL: 3
	RTL: 1	SRL: 1	RTL: 1	RTL: 1
west approach	LTL: 2	SLL: 1	LTL: 1	LTL: 1
	`TL: 2		TL: 3	TL: 3
	RTL: 1	SRL: 1	RTL: 1	RTL: 1
**Channelization (Lane Number Increase)**				
north approach	2 → 4 lanes	2 → 3 lanes	2 →3 lanes	5 → 7 lanes
south approach	3 → 4 lanes	No	2 → 3 lanes	4 → 6 lanes
east approach	4 → 5 lanes	No	No	4 → 5 lanes
west approach	4 → 5 lanes	No	4 → 5 lanes	3 → 5 lanes
**Guardrail Installation**				
north approach	No	Yes	No	Yes
south approach	Yes	Yes	No	Yes
east approach	No	No	Yes	Yes
west approach	Yes	No	Yes	Yes
**Traffic Enforcement Camera Location**				
northbound	Yes	Yes	Yes	Yes
southbound	No	Yes	Yes	No
eastbound	Yes	Yes	Yes	Yes
westbound	Yes	Yes	Yes	No

LTL represents the left-turn lane; TL stands for the through lane; RTL denotes the right-turn lane; SLL denotes from straight to left-turn lane; SRL stands for from straight to right-turn lane. → represents the increase of lane number. For example, 2→ 4 lanes means that the number of lanes at an intersection approach has increased from 2 to 4.

### Data collection and processing

A total of 13,008 records of traffic violations from May 1st to July 31st, 2018, were collected from traffic enforcement cameras, which saved and recorded the traffic violations in the form of screenshots and corresponding documents at the selected signalized intersections. The records included license plate number, vehicle type, occurrence position, occurrence time, and specific behavior of traffic violation. In the dataset, there were only four types of traffic violations, including WWD, RLR, VTM, and DIOL.

In order to mine more possible influence factors according to the collected dataset, the information mentioned above was further processed. Hence, the vehicle type was classified into small cars and other types. Given ownership, the vehicle included local and non-local vehicles. In the light of occurrence time, temporal factors were specifically expanded, and then categorized into time of day, day of week, and month.

Moreover, the weather conditions were considered and collected from the 2345 Weather Forecast website (http://tianqi.2345.com/wea_history/53463.htm) by using the occurrence location and time of traffic violations. The weather status and temperature (here refers to the average temperature) were obtained. The weather status consisted of sunny, cloudy, partly cloudy, and rainy. The temperature was categorized into three groups: less than 10 degrees centigrade, from 11 to 20 degrees centigrade, and between 21 and 30 degrees centigrade.

## Preliminary analysis

### Characteristics of traffic violations by vehicle conditions

The number of traffic violations by vehicle type and ownership at each signalized intersection is shown in Table 2. It is observed that small cars and local vehicles had a higher proportion of traffic violations than other vehicle types and non-local vehicles at four signalized intersections. Besides, both small cars and other types of vehicles at I1 had more RLR behaviors. So were the local and non-local vehicles at I1. Small cars, local and non-local vehicles at I2, I3, and I4 had more VTM than others. Meanwhile, for local and non-local vehicles, DIOL at I3 was relatively common.

**Table 2:** Number of traffic violations by vehicle type and ownership.

Condition	Traffic violation	Four selected signalized intersections			
		I1	I2	I3	I4
**Vehicle type**					
Small car	WWD	1	20	55	16
	RLR	296	425	132	248
	VTM	14	907	442	9905
	DIOL	93	46	214	188
Others	WWD	0	0	0	0
	RLR	1	0	0	0
	VTM	0	1	0	3
	DIOL	0	0	1	0
**Vehicle ownership**					
Local vehicle	WWD	1	17	50	15
	RLR	259	370	107	210
	VTM	10	831	360	9447
	DIOL	71	41	172	136
Non-local vehicle	WWD	0	3	5	1
	RLR	38	55	25	38
	VTM	4	77	82	461
	DIOL	22	5	43	52

### Characteristics of traffic violations by temporal distributions

**Fig 3:**
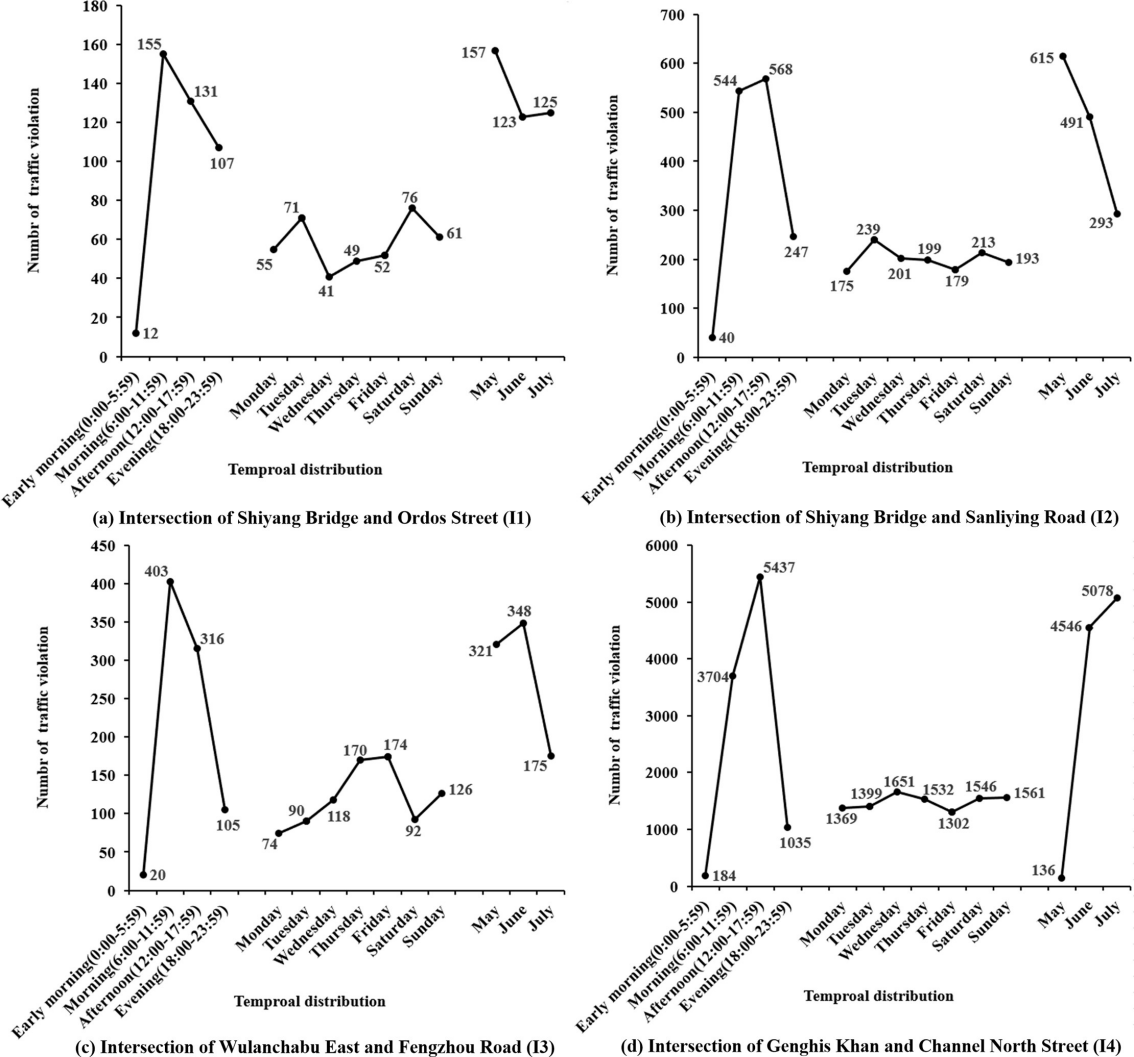
Time distribution of traffic violations.

The number of traffic violations by temporal distributions at four signalized intersections is presented in Fig 3. As for time of day, traffic violations at four intersections mainly occurred in the morning (6:00–11:59) and afternoon (12:00–17:59), while rarely happened in the early morning (0:00–5:59). With regard to day of week, traffic violations at I1 primarily concentrated on Tuesday and weekend. Traffic violations at I3 frequently occurred on Thursday and Friday, whereas at I2 and I4 evenly distributed within a week. As to month, the distribution of traffic violations at four signalized intersections was quite different. The number of traffic violations at I1 had little difference in three months. At I3, it was relatively stable in the first two months but greatly reduced in July. The number of traffic violations at I2 apparently decreased with the month. Inversely, the number of traffic violations at I4 evidently increased with the month, especially in June and July. It may be related to the actuality that the I4 has more approaches in each direction.

### Characteristics of traffic violations by weather conditions

**Fig 4:**
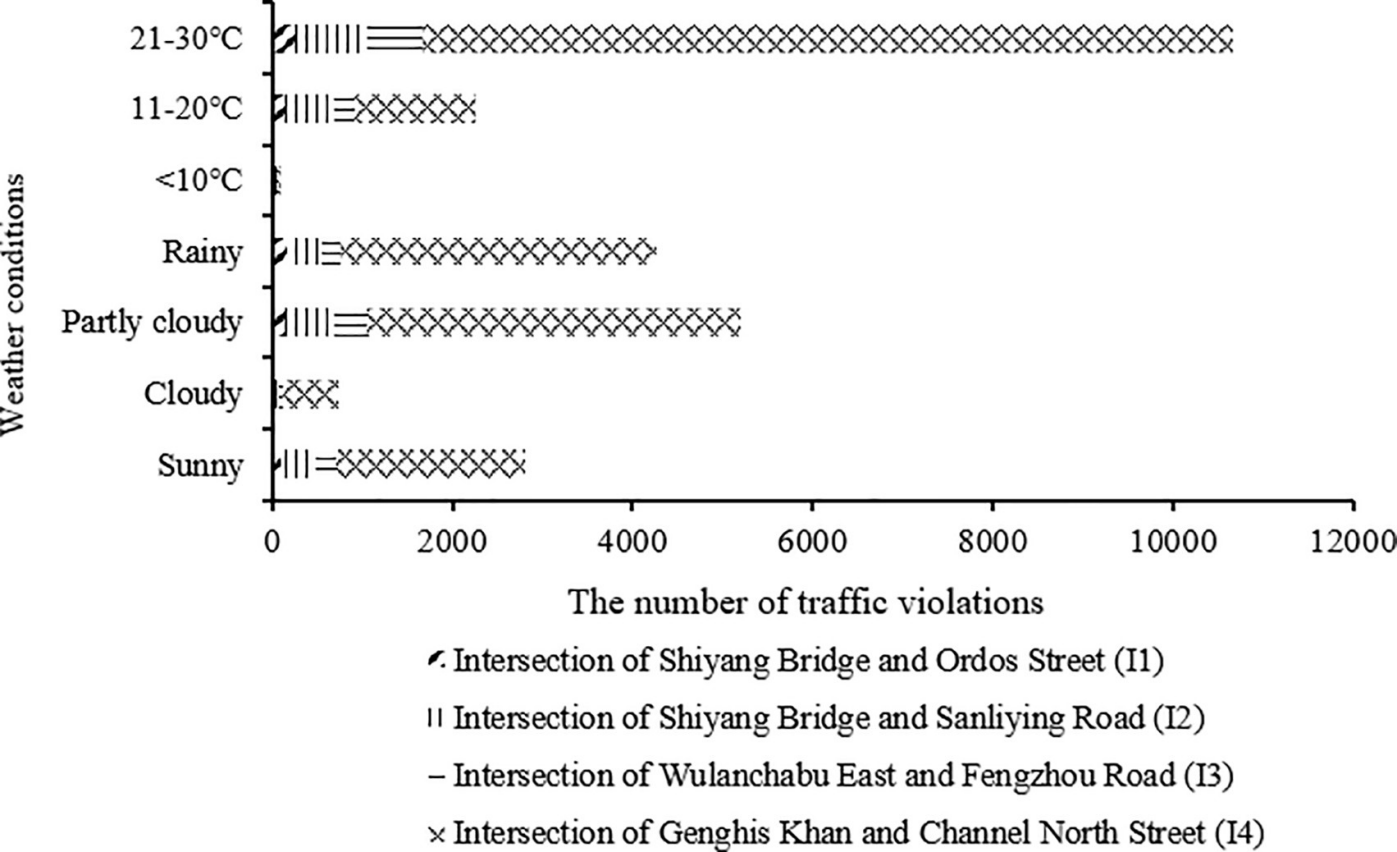
Number of traffic violations under various weather conditions.

The number of traffic violations under various weather conditions is shown in Fig 4. In regard to weather status, it indicated that most violations happened on partly cloudy day, following by on rainy, sunny, and cloudy day. With respect to temperature, most drivers committed violations under the temperature between 21 and 30 degrees centigrade, following by under the temperature from 11 to 20 degrees centigrade and less than 10 degrees centigrade. Moreover, traffic violations primarily occurred at I4 no matter what the weather status and temperature were. Dissimilarly, the number of traffic violations at I2 and I3 was approximately alike under each condition, while at I1 was the smallest.

## Modeling analysis

### Methodology

In this study, the OMLM was developed to investigate the influence factors of signalized intersection traffic violations, with the DIOL as the reference level.

The OMLM is expressed as follows [35, 36]:

$$P_{n}(Y=i)=\frac{EXP\left(\alpha_{i}+\beta_{ik}x_{ik}\right)}{\underset{\forall I}{\sum }EXP\left(\alpha_{I}+\beta_{Ik}x_{Ik}\right)}$$ (1)

Where, *Y* denotes the dependent variable with *I* categories; *P*_*n*_(*Y* = *i*) is the probability of observation *n* having the discrete outcome *i*, *i*∈*I*; *α*_*i*_ represents the intercept corresponding to the outcome *i*; *x*_*ik*_ is the *k*th independent variable corresponding to the outcome *i*; *β*_*ik*_ denotes the *k*th regression parameter corresponding to outcome *i*, which is estimated by using maximum likelihood estimation.

The OMLM can be rewritten as:

$$\log\left[\frac{P_{n}(Y=i)}{P_{n}(Y=I)}\right]=\alpha_{i}+\beta_{ik}x_{ik}$$ (2)

Where, *P*_*n*_(*Y* = *I*) is the probability of observation *n* having the baseline category *I*.

Although ordinary multinomial logit models have been widely applied during the past years, people found some limitations of this model, such as, not considering observed and unobserved heterogeneity in parameter effects [37, 38]. Since the data were collected at different approaches of four signalized intersections, the REMLM was necessarily adopted to account for the unobserved heterogeneity between observations. Then, the REMLM is expressed as [39]:

$$\log\left[\frac{P_{n}(Y=i)}{P_{n}(Y=I)}\right]=\alpha_{i}+\beta_{ik}x_{ik}+v_{ni}$$ (3)

Where, *v*_*ni*_ is the variable of random effects between observations assumed to be distributed as $N(0,\sigma_{vi}^{2})$. The parameters in REMLM were estimated by using Markov Chain Monte Carlo sampling.

The Stata 15 was applied to run OMLM and REMLM. In both models, the independent variables included vehicle factors, temporal factors, road conditions, traffic enforcement and management facilities, and weather conditions. All these independent variables were categorical variables. Descriptions of independent variables are presented in Table 3.

**Table 3:** Descriptions of independent variables.

Independent variable	Descriptions	Mean	Standard error
**Vehicle factor**			
Type	Small car-1, others-0	0.00046	0.02147
Ownership	Local-1, non-local-0	0.07003	0.25521
**Temporal factor**			
Time of day	Early morning (0:00–5:59)-1	0.98032	0.13890
	Morning (6:00–11:59)-2	0.63053	0.48267
	Afternoon (12:00–17:59)-3	0.66666	0.51639
	Evening (18:00–23:59)-4	0.88515	0.31886
Day of week	Monday-1	0.86785	0.33867
	Tuesday-2	0.84178	0.36646
	Wednesday-3	0.85055	0.35654
	Thursday-4	0.86747	0.33908
	Friday-5	0.85632	0.35078
	Saturday-6	0.84809	0.35894
	Sunday-7	0.86954	0.33682
Month	May-1	0.85947	0.34755
	June-2	0.57657	0.49412
	July-3	0.56404	0.49590
**Road and Traffic Management Conditions**			
Left-turn lane ratio	26%-1	0.20356	0.40267
	27%-2	0.93511	0.24633
	28%-3	0.10755	0.30982
	40%-4	0.89245	0.30982
Channelization ratio	25%-1	0.89245	0.30982
	75%-2	0.93512	0.24633
	100%-3	0.17243	0.37777
Guardrail ratio	75%-1, 100%-0	0.20357	0.40267
Enforcement camera ratio	50%-1	0.20357	0.40267
	75%-2	0.96887	0.17368
	100%-3	0.82757	0.37777
**Weather**			
Status	Sunny-1	0.78444	0.41123
	Cloudy-2	0.94326	0.23124
	Partly cloudy-3	0.60078	0.48976
	Rainy-4	0.67152	0.46968
Temperature	<10°C-1	0.99292	0.08381
	11~20°C-2	0.71584	0.37830
	21~30°C-3	0.18011	0.38430

Moreover, McFadden pseudo *R*^2^ and AIC (Akaike Information Criterion) were adopted to compare OMLM and REMLM. The McFadden *ρ*^2^ is given by [36]:

$$pseudo\;R^{2}=1-LL_{\beta }/LL_{0}$$ (4)

Where, *LL*_*β*_ is the log-likelihood at convergence with parameters; *LL*_0_ is the log-likelihood at convergence without parameters.

The AIC is defined as:

$$AIC=-2LL_{\beta }+2q$$ (5)

Where, *q* is the number of estimated parameters. The smaller AIC value indicates a better-fitted model[40].

In addition, the marginal effects of independent variables were calculated to interpret the influences of estimated coefficients on the probabilities of all categories of the dependent variables. In this study, all independent variables were indicator variables. Hence, the marginal effects were calculated as the difference in the estimated probabilities with the indicator variable varying from zero to one, whereas all other variables are equal to their means[36]. The marginal effects are given by:

$$\Delta P_{n}(Y=i)=P_{nx_{ik}=1}(Y=i)-P_{nx_{ik}=0}(Y=i)$$ (6)

### Results of model estimation

The estimation results of OMLM and REMLM of WWD, RLR and VTM are presented in Table 4, Table 5, and Table 6, respectively. According to the McFadden *pseudo R*^2^ and AIC values listed in Tables 4–6, the REMLM outperformed the OMLM (5009.102 *vs* 5340.518). All the estimated parameters included in both models were statistically significant at the 90 percent confidence interval at least. It is observed that the two models shared similar significant factors, including vehicle factor, temporal factors, road characteristics, and weather conditions. It is noteworthy that the effect of channelization ratio on traffic violations was not ignored, although it was only significant in OMLM. The channelization ratio of 100% evidently increased the probabilities of WWD and VTM, while decreased the probability of RLR.

**Table 4:** Estimation results of OMLM and REMLM of WWD.

Explanatory variables	OMLM			REMLM	
	Coef.	Std. Dev.	OR	Coef.	MCSE
**Intercept**	20.710	1.444	NA	-15.956	0.107
**Vehicle factor**					
Local vehicle	-1.614***	0.396	0.199	-1.708***	0.017
**Time of Day**					
Morning (6:00–11:59)	1.761***	0.519	5.817	2.028**	0.073
Afternoon (12:00–17:59)	2.814***	0.521	8.880	2.430***	0.048
Evening (18:00–23:59)	2.091***	0.577	8.093	2.374***	0.028
**Day of Week**					
Tuesday	0.238*	0.444	1.269	0.235*	0.032
Wednesday	0.343*	0.450	1.408	0.293*	0.029
Thursday	1.606***	1.587***	0.015	0.170*	0.008
Friday	0.152*	0.373*	0.041	0.283*	0.009
Saturday	0.527*	0.572*	0.038	0.371**	0.008
Sunday	-0.114*	-0.063*	0.017	0.077*	0.009
**Month**					
June	-1.097***	-1.028***	0.016	-0.639***	0.013
July	-1.538***	-1.480***	0.012	-1.507***	0.012
**Road condition**					
Left-turn lanes ratio of 26%	-2.069**	-1.753**	0.022	-5.764***	0.007
Left-turn lanes ratio of 28%	-3.169***	-3.471***	0.020	-2.987***	0.010
Left-turn lanes ratio of 40%	-3.700***	-4.002***	0.445	-5.359***	0.014
**Traffic management facility**					
Channelization ratio of 100%	3.169***	NA	NA	NA	NA
**Weather status**					
Cloudy	0.958*	1.006*	0.034	0.704***	0.008
Partly cloudy	0.363*	0.339*	0.024	-0.008*	0.008
Rainy	0.517*	0.475*	0.028	0.153*	0.009
**Temperature**					
< 10°C	NA	14.052**	0.048	1.213***	0.013
21~30°C	-0.527*	-0.515*	0.013	-0.122*	0.010
**Model statistics**					
Number of observations	13008			13008	
-2 Log-likelihood	5298.518			4967.102	
Mcfadden’s *pseudo R*^*2*^	0.325			0.367	
AIC	5340.518			5009.102	

* Represents significance at 90% level

** denotes significance at 95% level

***represents significane at 99% level; MCSE represents the Monte-Carlo standard error; NA denotes not applicable.

**Table 5:** Estimation results of OMLM and REMLM of RLR.

Explanatory variables	OMLM			REMLM	
	Coef.	Std. Dev.	OR	Coef.	MCSE
**Intercept**	-4.996	1.311	NA	-4.955	0.009
**Vehicle factor**					
Local vehicle	-0.599***	0.148	0.572	-0.612***	0.022
**Time of Day**					
Morning (6:00–11:59)	0.858***	0.369	2.358	0.851***	0.017
Afternoon (12:00–17:59)	1.347***	0.367	3.846	1.368***	0.015
Evening (18:00–23:59)	1.261***	0.380	3.529	1.276***	0.012
**Day of Week**					
Tuesday	0.483**	0.227	1.621	0.433**	0.014
Wednesday	0.063*	0.230	1.065	0.051*	0.011
Thursday	0.531**	0.219	1.701	0.498**	0.015
Friday	0.557**	0.231	1.746	0.561**	0.010
Saturday	0.616***	0.217	1.852	0.618***	0.012
Sunday	0.295*	0.232	1.343	0.300*	0.005
**Month**					
June	0.251*	0.167	1.286	0.232*	0.011
July	-0.276*	0.208	0.759	-0.240*	0.012
**Road condition**					
Left-turn lane ratio of 26%	0.884***	0.153	2.421	0.786***	0.014
Left-turn lane ratio of 28%	1.649***	0.162	5.202	1.559***	0.011
Left-turn lane ratio of 40%	-1.062***	0.195	0.346	-1.183***	0.018
**Traffic management facility**					
Channelization ratio of 100%	-1.649***	0.162	0.192	NA	NA
**Weather status**					
Cloudy	0.181*	0.277	1.198	0.160*	0.010
Partly cloudy	-0.449***	0.156	0.638	-0.420***	0.010
Rainy	-0.301**	0.174	0.740	-0.264**	0.008
**Temperature**					
< 10°C	0.701**	0.383	2.015	0.717**	0.015
21~30°C	0.005*	0.162	1.005	0.041*	0.010
**Model statistics**					
Number of observations	13008			13008	
-2 Log-likelihood	5298.518			4967.102	
Mcfadden’s *pseudo R*^*2*^	0.325			0.367	
AIC	5340.518			5009.102	

* Represents significance at 90% level

** denotes significance at 95% level

***represents significane at 99% level; MCSE represents the Monte-Carlo standard error; NA denotes not applicable.

**Table 6:** Estimation results of OMLM and REMLM of VTM.

Explanatory variables	OMLM			REMLM	
	Coef.	Std. Dev.	OR	Coef.	MCSE
**Intercept**	0.865	1.326	NA	10.916	0.013
**Vehicle factor**					
Local vehicle	-1.280***	0.132	0.278	-1.299***	0.007
**Time of Day**					
Morning (6:00–11:59)	-0.172*	0.357	0.842	-0.116*	0.011
Afternoon (12:00–17:59)	0.112*	0.354	1.118	0.181*	0.015
Evening (18:00–23:59)	0.147*	0.367	1.158	0.233*	0.005
**Day of Week**					
Tuesday	-0.009*	0.205	0.991	-0.067*	0.015
Wednesday	-0.166*	0.208	0.847	-0.197*	0.007
Thursday	0.195*	0.196	1.216	0.170*	0.008
Friday	0.274*	0.206	1.315	0.283*	0.009
Saturday	0.355**	0.197	1.426	0.371**	0.008
Sunday	0.102*	0.211	1.107	0.077*	0.009
**Month**					
June	-0.618***	0.148	0.539	-0.639***	0.013
July	-1.493***	0.188	0.225	-1.507***	0.012
**Road condition**					
Left-turn lane ratio of 26%	-5.858***	0.296	0.003	-5.764***	0.007
Left-turn lane ratio of 28%	-2.614***	0.298	0.073	-2.987***	0.010
Left-turn lane ratio of 40%	-4.876***	0.324	0.008	-5.359***	0.014
**Traffic management facility**					
Channelization ratio of 100%	2.614***	0.298	13.656	NA	NA
**Weather status**					
Cloudy	0.717***	0.228	2.047	0.704***	0.008
Partly cloudy	-0.043*	0.139	0.958	-0.008*	0.008
Rainy	0.115*	0.156	1.122	0.153*	0.009
**Temperature**					
< 10°C	1.127***	0.377	3.088	1.213***	0.013
21~30°C	-0.161*	0.144	0.851	-0.122*	0.010
**Model statistics**					
Number of observations	13008			13008	
-2 Log-likelihood	5298.518			4967.102	
Mcfadden’s *pseudo R*^*2*^	0.325			0.367	
AIC	5340.518			5009.102	

* Represents significance at 90% level

** denotes significance at 95% level

***represents significane at 99% level; MCSE represents the Monte-Carlo standard error; NA denotes not applicable.

Table 7 lists the marginal effects of each explanatory variables for the four types of signalized intersection traffic violations in the REMLM. If one vehicle was a local vehicle, the probabilities of it committing RLR and DIOL increased by 2.7% and 3.7%, respectively. On the contrary, the probabilities of this type of vehicle committing WWD and VTM decreased by 0.4% and 5.9%, respectively.

**Table 7:** Marginal effects of explanatory variables in REMLM.

Explanatory variables	Probability of four types of traffic violation			
	WWD	RLR	VTM	DIOL
**Vehicle factor**				
Local vehicle	-0.004	0.027	-0.059	0.037
**Time of day**				
Morning (6:00–11:59)	0.012	0.051	-0.057	-0.005
Afternoon (12:00–17:59)	0.013	0.065	-0.060	-0.017
Evening (18:00–23:59)	0.012	0.059	-0.054	-0.017
**Day of week**				
Tuesday	0.001	0.026	-0.023	-0.005
Wednesday	0.003	0.010	-0.016	0.003
Thursday	0.009	0.018	-0.016	-0.011
Friday	-0.001	0.018	-0.005	-0.012
Saturday	0.001	0.017	-0.004	-0.015
Sunday	-0.002	0.012	-0.005	-0.005
**Month**				
June	-0.005	0.041	-0.050	0.013
July	-0.003	0.051	-0.087	0.039
**Road condition**				
Left-turn lane ratio of 26%	0.013	0.294	-0.434	0.127
Left-turn lane ratio of 28%	-0.012	0.215	-0.258	0.055
Left-turn lane ratio of 40%	-0.002	0.169	-0.304	0.137
**Weather status**				
Cloudy	0.003	-0.023	0.015	0.005
Partly cloudy	0.003	-0.022	0.039	-0.019
Rainy	0.003	-0.022	0.019	0.000
**Temperature**				
< 10°C	0.088	-0.027	-0.014	-0.047
21~30°C	-0.003	0.008	-0.009	0.004

With respect to time of day, in the morning (6:00–11:59), the probabilities of vehicles driving in wrong way and running red light increased by 1.2% and 5.1%, respectively. However, the probabilities of vehicles committing VTM and DIOL decreased by 5.7% and 0.5%, respectively. Similarly, in the afternoon (12:00–17:59), the probabilities of vehicles driving in wrong way and running red signal increased by 1.3% and 6.5%, respectively. Nevertheless, the probabilities of vehicles committing VTM and DIOL reduced by 6% and 1.7%, respectively. When it was in the evening (18:00–23:59), the probabilities of vehicles driving in wrong way and running red light rose by 1.2% and 5.9%, respectively. Nonetheless, the probabilities of vehicles committing VTM and DIOL declined by 5.4% and 1.7%, respectively.

In terms of day of week, Tuesday, Wednesday, Thursday, and Saturday were related to the higher likelihoods of WWD and RLR, but the lower likelihoods of VTM and DIOL. Friday and Sunday were associated with the higher probability of RLR, while the lower probabilities of WWD, VTM, and DIOL.

With regard to monthly difference, in June, the probabilities of vehicles committing RLR and DIOL ascended by 4.1% and 1.3%, respectively. Nevertheless, the probabilities of vehicles committing WWD and VTM descended by 0.5% and 5%, respectively. Similarly, in July, the likelihoods of vehicles committing RLR and DIOL increased by 5.1% and 3.9%, respectively. Nonetheless, the likelihoods of vehicles committing WWD and VTM decreased by 0.3% and 8.7%, respectively.

As for specific road conditions, the left-turn lane ratio of 26% increased the probabilities of WWD, RLR, and DIOL occurrence by 1.3%, 29.4%, and 12.7%, respectively, whereas decreased the probability of VTM occurrence by 43.4%. The left-turn lane ratio of 28% increased the likelihoods of RLR and DIOL occurrence by 21.5% and 5.5%, respectively, while decreased the likelihoods of WWD and VTM occurrence by 1.2% and 25.8%, respectively. The left-turn lane ratio of 40% increased the probabilities of RLR and DIOL happening by 16.9% and 13.7%, respectively, whereas decreased the probabilities of WWD and VTM happening by 0.2% and 30.4%, respectively.

In terms of weather status, cloudy and rainy days increased the probabilities of WWD, VTM, and DIOL, while reduced the probability of RLR taking place. However, the partly cloudy days increased the likelihoods of WWD and VTM occurring, while reduced the probabilities of RLR and DIOL occurring.

As to temperature conditions, under the temperature of less than 10 degrees centigrade, the likelihood of vehicles committing WWD ascended by 8.8%, while the likelihoods of vehicles committing RLR, VTM, and DIOL descended by 2.7%, 1.4%, and 4.7%, respectively. Dissimilarly, under the temperature of from 21 to 30 degrees centigrade, the likelihoods of WWD and VTM occurrence reduced by 0.3% and 0.9%, respectively, whereas the probabilities of RLR and DIOL occurrence increased by 0.8% and 0.4%, respectively.

## Discussions

The outcomes of this study manifested that local vehicle, time of day, day of week, month, left-turn lane ratio, channelization ratio, weather status, and temperature had varying influences on four types of traffic violations at signalized intersections.

Unlike the previous studies [41, 42], our data displayed that vehicle type did not exhibit a significant effect on traffic violations at signalized intersections. However, vehicle ownership was found to impact signalized intersection traffic violations significantly. As compared to local vehicles, non-local vehicles were more likely to commit WWD and VTM. This may be ascribed to non-local drivers’ unfamiliarity with road conditions, or road lack of traffic control devices such as signs and pavement markings [43].

It was found that time of day affected signalized intersection traffic violations to a certain extent. The drivers were more likely to commit WWD and RLR in the morning (6:00–11:59), afternoon (12:00–17:59), and evening (18:00–23:59). Duing nighttime hours, poor lighting conditions, and lack of signage and pavement markings are probably the causes of WWD [44]. Furthermore, this finding supports the uneven distribution of traffic violations over time of day in the literature [41]. The differences regarding RLR distribution over time of day in this study demonstrate the findings in one previous research [12]. In the morning (6:00–11:59) and afternoon (12:00–17:59), it is probably because of that sunlight reduces the visibility of the color of signal lights, which leads to RLR [41]. In the evening (18:00–23:59), bad visibility may also cause RLR. Additionally, RLR is more likely to happen in the evening (18:00–23:59) than in the morning (6:00–11:59) [15].

The findings of this study indicated that there existed obvious differences in the distribution of four types of traffic violations over day of week. Consistent with previous studies[21, 41, 45], RLR was more likely to occur on the weekend. Moreover, RLR was more likely to happen from Tuesday to Friday than on Monday. High-frequency occurrence and being detected probably may be on account of mild penalties and weak enforcement. Similarly, WWD frequently took place on Tuesday, Wednesday, Thursday, and Saturday.

Interestingly, RLR and DIOL were more likely to occur in June and July. This probably was related to high temperatures in these two months. Furthermore, our results also revealed that the likelihoods of vehicles committing RLR and DIOL under the temperature from 21 to 30 degrees centigrade were much higher than that under the temperature between 11 and 20 degrees centigrade. The drivers may be easily irritable under the high temperature condition and become impatient to wait for green lights at signalized intersections. And thus, they may either run red lights or drive in an inaccurate oriented lane to pass through the signalized intersection. A number of studies have verified the significant effects of bad emotion on traffic violations [22, 46, 47].

Similarly, weather status was found to significantly impact the likelihoods of WWD, RLR, VTM, and DIOL occurrence. On cloudy, partly cloudy, and rainy days, the likelihoods of vehicles committing WWD and VTM apparently increased. These findings are in accord with the results of existing literature [22, 41], showing that bad weather significantly increases traffic violations.

It was also found that the left-turn lane ratio was associated with traffic violations at signalized intersections. Unlike the previous study[48], exclusive left-turn lanes are commonly used as a common traffic engineering measure to reduce conflicts with through traffic. In our study, left-turn lane ratio evidently increased RLR and DIOL, whereas distinctly decreased WWD and VTM. This may be by reason of the situation where, with the increase in the left-turn lane ratio, the drivers have more lane-specific conditions and opportunities to run red lights and drive in the inaccurate oriented lanes.

In addition, the estimation results of OMLM indicated that the channelization ratio obviously influenced traffic violations at signalized intersections. In other words, adding the number of lanes at the entry of signalized intersection approaches could increase WWD and VTM, while decrease RLR. As approaching the intersection, the number of lanes in one traveling direction increasing, the drivers may either cross the solid line when they find themselves traveling in a wrong oriented lane or travel over the solid line. Likewise, under such a situation, the drivers may commit WWD.

It should be noted that the relationships between traffic violations and influence factors reflected by the estimated coefficients of some factors in REMLM are opposite to that reflected by the marginal effects. This reason is that REMLM needs to set one type of traffic violation as the baseline category, which makes what the coefficients reflect is the changes in each outcome probability relative to the baseline category probability. In other words, the estimated coefficients may magnify, reduce, or even reverse the effects of every single factor. However, such issues could be avoided by calculating the marginal effects of influence factors [49]. Accordingly, the marginal effects of influence factors in the REMLM were employed to interpret the relationships between traffic violations and contributing factors.

The findings of current study imply that traffic violation intervention at signalized intersection should target different vehicle types and consider temporal factors, road, and weather conditions. As to non-local vehicles, they should be provided with more guidances at intersection approaches, such as traffic marking systems, sound intelligent traffic guidance sign systems, and other mass media to develop education and awareness programs[43]. Besides, visual intervention also has a signifcant influence on drivers’ behavior [50]. Since the WWD and RLR frequently happen in the daytime and evening (6:00–23:59), and most days within one week, the violators should be confronted with tougher penalties and enforcements.

Additional measures targeted RLR and DIOL during June and July are also needed, such as increasing traffic policemen at signalized intersections. As left-turn lane ratio significantly increases RLR and DIOL, an appropriate number of left-turn lanes should be considered during the process of intersection layout design. In addition, channelization ratio (i.e., increasing lane number at intersection approaches) was found to increase WWD and VTM evidently. Hence, if the lane number at one intersection approach is increased, WWD dynamic warning sign [51], improved pavement markings [26], as well as guardrail installation at the corresponding intersection exit is recommended to prevent WWD; traffic oriented arrow ahead, smart traffic markings [25], and colored pavement[52] are suggested to deter VTM.

Moreover, since the adverse weather status was found to increase WWD and VTM distinctly, additional measures to improve the legibility under the cloudy, partly cloudy, and rainy days are required as well. The hot weather ruins drivers’ mood and withdraws attention from the driving related information[22], and then induces unsafe driving behaviors, like RLR and DIOL in this study. Accordingly, educational programs regarding the consequences of negative emotional responses to hot weather should be conducted to target RLR and DIOL violators. Furthermore, psychological interventions should teach these drivers on how to effectively regulate emotions under the hot weather condition and how to comply with traffic lights and oriented lane guidance regulations.

## Conclusions

In this paper, a total of 13,008 records of traffic violations in Hohhot from May 1st to July 31st, 2018, were collected from traffic enforcement cameras. In our dataset, there were four traffic violations, namely RLR, WWD, VTM, and DIOL. After preliminarily analyzing the characteristics of traffic violations at signalized intersections by road geometric, traffic enforcement and management measures, vehicle and temporal attributes, and weather condition, the ordinary multinomial logit model (OMLM) was developed to uncover the effects of contributing factors to traffic violations. By considering the unobserved heterogeneity between observations, a random effects multinomial logit model was proposed as well, which outperformed the former. At the same time, the marginal effects of independent variables were applied to figure out the extent to which a particular factor affected traffic violations. The major conclusions obtained from this study are summarized as follows:

- Non-local vehicles were more likely to commit WWD and VTM than local vehicles.
- WWD and RLR frequently occurred in the daytime and evening (6:00–23:59), and on most days within a week.
- RLR and DIOL mainly happened in June and July.
- The left-turn lane ratio significantly increased RLR and DIOL.
- The cloudy, partly cloudy, and rainy days obviously increased WWD and VTM.
- The temperature from 21 to 30 degrees centigrade was apparently associated with the higher likelihoods of RLR and DIOL.
- Some intervention measures, targeting different vehicle types and considering temporal factors, road, and weather conditions, were recommended to reduce WWD, RLR, VTM, and DIOL at signalized intersections.

However, there exist some limitations in the present study. Although the dataset used in the current study is gathered from traffic enforcement cameras at signalized intersections, it contains very limited information. First, some other possible traffic conditions, road, and environmental factors of these four types of traffic violations cannot be explored. This can be addressed with more enriched data. Second, our dataset does not include some other intersection traffic violations, such as mobile phone usage, failure to wear a seatbelt, and pedestrian violations. Whether the factors identified by this study also influence these violations cannot be determined. Future research work will detect these violations and appraisal their corresponding influence factors based on the data collected by novel technologies and methods, like using video sensors [53]. Third, spatial factors cannot be examined because of the collected data only from four signalized intersections in Hohhot, so that it is difficult to uncover the relationship between traffic violations and spatial factors. Fourth, since the data is only from May 1st to July 31st, the influences on traffic violations of weather conditions during the whole year cannot be investigated. Last but not the least, in current research, each traffic violation type is not separated for further analysis. The relationship between left-turn lane ratio and left-turn RLR and go-through RLR needs to be further investigated, respectively. The VTM needs further investigation on violations of lane change and driving over lane line. Moreover, the DIOL can be classified into nine classifications[25]. Consequently, the influence factors of type-specific traffic violations at signalized intersections merit further studies.
